# Virucidal Activity of Different Mouthwashes Using a Novel Biochemical Assay

**DOI:** 10.3390/healthcare10010063

**Published:** 2021-12-30

**Authors:** Héctor J. Rodríguez-Casanovas, Manuel De la Rosa, Yesit Bello-Lemus, Giulio Rasperini, Antonio J. Acosta-Hoyos

**Affiliations:** 1 Independent Researcher, 35004 Gijón, Spain; 2 Department of Periodontics, AME University Monterrey, Monterrey 64060, Mexico; drmanuel@drmdelarosa.com; 3 School of Basic and Biomedical Sciences, Universidad Simón Bolívar, Barranquilla 080002, Colombia; yesit.bello@unisimonbolivar.ed.co; 4 Department of Biomedical, Surgical and Dental Sciences, University of Milan, 20122 Milan, Italy; giulio@studiorasperini.it; 5 Department of Periodontics and Oral Medicine, School of Dentistry, University of Michigan, Ann Arbor, MI 48109, USA

**Keywords:** mouthwash, SARS-CoV-2, virucidal activity, viral load, COVID-19, CPC, D-limonene, antiseptic

## Abstract

Background: Saliva of patients with COVID-19 has a high SARS-CoV-2 viral load. The risk of spreading the virus is not insignificant, and procedures for reducing viral loads in the oral cavity have been proposed. Little research to date has been performed on the effect of mouthwashes on the SARS-CoV-2 virus, and some of their mechanisms of action remain unknown. Methods: SARS-CoV-2 positive nasopharyngeal swabs measured by RT-PCR were used for virucidal activity in a 1:1 ratio, with an incubation time of 1 min. The solutions used in this study were: iodopovidone (8 mg); * D-limonene, a terpene extracted from citrus peels (0.3%); ^†^ cetylpyridinium chloride (0.1%) (CPC); ^‡^ chlorhexidine gluconate (10%) (CHX); ^§^ a CPC (0.12%) and CHX (0.05%) containing formula; ** a formula containing essential oils; ^††^ a CPC containing formula (0.07%); ^‡‡^ a D-limonene (0.2%) and CPC (0.05%) containing formula; ^§§^ a solution containing sodium fluoride (0.05%) and CPC (0.075%); *** a solution containing CHX (0.12%) and; ^†††^ a CHX (0.2%) containing formula. ^‡‡‡^ As a control reaction, saline solution or excipient solution (water, glycerin, citric acid, colorant, sodium citrate) was used. Conclusion: Within the limitations of this study, we can conclude that a mouthwash containing both D-limonene and CPC reduced the virucidal activity in about 6 logs (>99.999% reduction). Hence, establishing a clinical protocol for dentists is suggested, where all patients to be treated rinse pre-operatively with a mouthwash containing both D-limonene and CPC to reduce the likelihood of infection with SARS-CoV-2 for dentists. This is a relatively inexpensive way to reduce viral transmission of SARS-CoV-2 from infected individuals within the community. It is also a simple way to decrease infections from asymptomatic and pre-symptomatic patients.

## 1. Introduction

Coronavirus disease 2019 (COVID-19) due to infection by severe acute respiratory syndrome coronavirus 2 (SARS-CoV-2) has spread around the world from December 2019 and by March 2020 was declared a pandemic by the World Health Organization (WHO). Worldwide, as of 7:07 pm CEST, 9 August 2021, there have been 202,608,306 confirmed cases of COVID-19, including 4,293,591 deaths, as reported by WHO [1].

Severe acute respiratory syndrome-related coronavirus (SARS-CoV-2) is a non-segmented positive-strand RNA enveloped virus and the agent responsible for causing coronavirus disease 19 (COVID-19). SARS-CoV-2 particles have a spherical shape, with diameters ranging from 60–140 nm, and are coated with 9–12 nm spike proteins in conjunction with the envelope and membrane proteins, which are embedded in a lipid bilayer viral envelope [2]. Inside the envelope, the single-stranded positive-sense viral RNA is associated with nucleocapsid proteins [3]. Possible ways to reduce transmission include targeting viral components, such as the membrane (envelope), surface spike proteins, and nucleic acids, to inactivate viruses.

Evidence shows that SARS-CoV-2 may be transmitted through contact, [4] airborne [5], fomite [6], fecal-oral [7], bloodborne [8], mother-to-child [7,9], and animal-to-human [10,11,12]. Infection with SARS-CoV-2 primarily causes respiratory illness, ranging from mild disease to severe disease and death, and some people infected with the virus are asymptomatic [13].

Since the beginning of the coronavirus pandemic, the main theory on viral transmission via small airborne micro-droplets (also commonly referred to as ‘aerosols’) has been intensely discussed in the context of the SARS-CoV-2/COVID-19 [13]. Several hospital-based studies have performed air sampling for SARS-CoV-2. At least four of these studies found several positive samples for SARS-CoV-2 genome (RNA) in the air using polymerase chain reaction (PCR) testing [14,15,16,17].

The cellular receptor for SARS-CoV-2 is angiotensin-converting enzyme II (ACE2). Because of this, ACE2-expressing cells are target cells and are susceptible to infection. ACE2 receptors are very common in the oral cavity and may be at a potential high-risk route for SARS-CoV-2 infection [18].

The virus can be identified in saliva even before COVID-19 symptoms occur, indicating that asymptomatic persons are at risk of virus transmission. Since transmission via salivary droplets or aerosols is the main way to spread SARS-CoV-2, reducing oral viral loads would be paramount in helping to control the spread of the virus. Dentists are, by definition, at a high risk of contracting infectious diseases. Special precautions, in addition to ordinary precautions, could prevent disease transmission from asymptomatic carriers. These extra precautions would not only aid in the control of COVID-19, but they would also serve as a guide for dealing with other respiratory illnesses [19]. Nevertheless, there is a study where it was concluded that the risk of transmission of SARS-CoV-2 and other respiratory viruses through aerosolized saliva in dental operatories is reasonably low, despite the limitations of a small sample size [20].

The salivary glands may be a major source of SARS-CoV-2 in saliva [21]. COVID-19 can be found in patients’ saliva at a rate of 91.7 percent, and the live virus can be grown in salivary samples [22]. This suggests that SARS-CoV-2 transmitted by asymptomatically infected individuals may originate from infected saliva [23].

Since the beginning of the pandemic, the use of antiseptic mouth rinses has been suggested to help diminish the risk of transmission of SARS-CoV-2 [24,25,26]. This might be extremely beneficial in dentistry in granting a more secure field to work in and also in daily routine for individuals using mouthwash regularly.

Currently, there is enough in vitro evidence to reinforce the use of mouthwashes to potentially reduce the viral load of SARS-CoV-2 and other coronaviruses [27].

New research to test this might include evaluating existing or specifically customized new formulations [25]. We developed a new biochemical assay to study the effect of mouthwash on the stability of the viral envelope and its ability to reduce the viral load. The aim of this study is to determine the virucidal activity of different mouthwashes using a novel biochemical assay.

## 2. Methods

### 2.1. Virus Samples and Products

SARS-CoV-2 positive nasopharyngeal swabs measured by RT-PCR with a virus concentration between 144,543 and 79,432,823 copies/mL (about 6–8 logs) were kept at −80 °C and used for the RNA inactivating assay in a 1:1 ratio with the inactivating solutions with incubation times of 1 min. The experiments were conducted with the same swab samples to ensure uniformity in the viral titers. The solutions used in this study are described in Table 1. As a control reaction, saline solution or excipient solution (water, glycerin, citric acid, colorant, sodium citrate) was used.

### 2.2. RNA Inactivating Assay

The indirect virucidal activity was determined by combining a previously quantified nasopharyngeal sample positive for SARS-CoV-2 with a mouthwash product or control for 1 min and shaking. Then, 1 U/mg of RNase A (Promega, Madison, WI, USA) was added and incubated for 1 min. The reaction was stopped by adding 400 μL of lysis solution, and the RNA was extracted with the Quick-DNA/RNA Viral MagBead Kit (Zymo Research, Irvine, CA, USA). An amount of 5 μL of the extracted solution was used for RT-PCR to measure the presence of SARS-CoV-2.

### 2.3. RT-qPCR

The real-time RT-qPCR protocol was adapted from the one used by Corman et al. [28]. Briefly, a 20 μL reaction was prepared containing 5 μL RNA, 400 nM primers, 200 nM probe, and 10 μL of 2 × reaction buffer provided with the iTaq universal Probe One-Step Kit (BioRad, Hercules, CA, USA). Oligonucleotides and probes targeting the E viral gene and human RNase P gene were synthesized and provided by LGC Biosearch Technologies (Petaluma, CA, USA). Thermal cycling was performed at 55 °C for 10 min for reverse transcription, followed by 95 °C for 3 min, and 45 cycles of 95 °C for 15 s and 58 °C for 30 s. To quantify the viral load, Cqs from the BioRad CFX96 system targeting the E gene were converted to viral load using a plasmid containing the sequence of SARS-CoV-2 genes E and RdRp, kindly provided by Jaime Castellanos’ Virology Laboratory, Universidad del Rosario, Colombia.

## 3. Results

**Figure 1:**
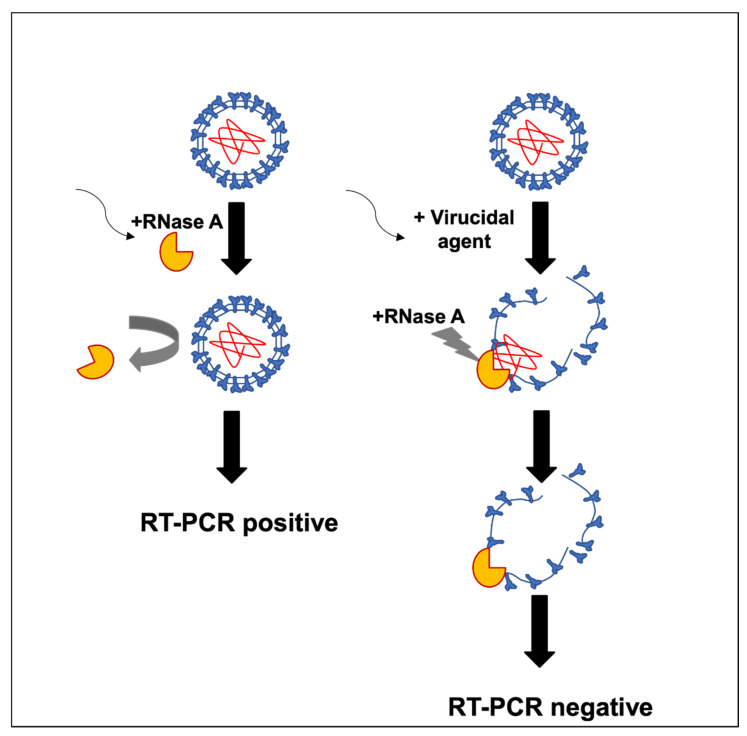
Model of the mechanism of action by mouthwash products on the viral envelope.

To evaluate the virucidal potential of different compounds and commercial mouthwash solutions, a method was designed to test the stability of the SARS-CoV-2 envelope to protect its RNA from RNase treatment directly from nasopharyngeal samples obtained from COVID-19 epidemiological surveillance. A model for the proposed mechanism of action by the mouthwash solutions on the viral envelope is described in Figure 1.

**Figure 2:**
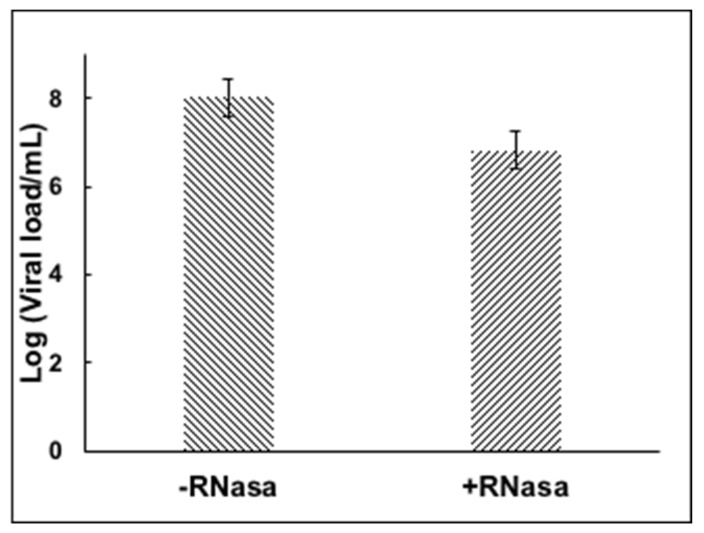
RNase treatment of nasopharyngeal sample containing SARS-CoV-2.

Previously diagnosed positive samples of SARS-CoV-2 RNA by RT-PCR were incubated in the presence of RNase to test whether enveloped viral particles, presumably infective, would protect the RNA from the enzymatic degradation. Figure 2 shows the stability of the viral RNA after the RNase treatment, suggesting the viral envelope protects the viral genome from enzymatic digestion.

**Figure 3:**
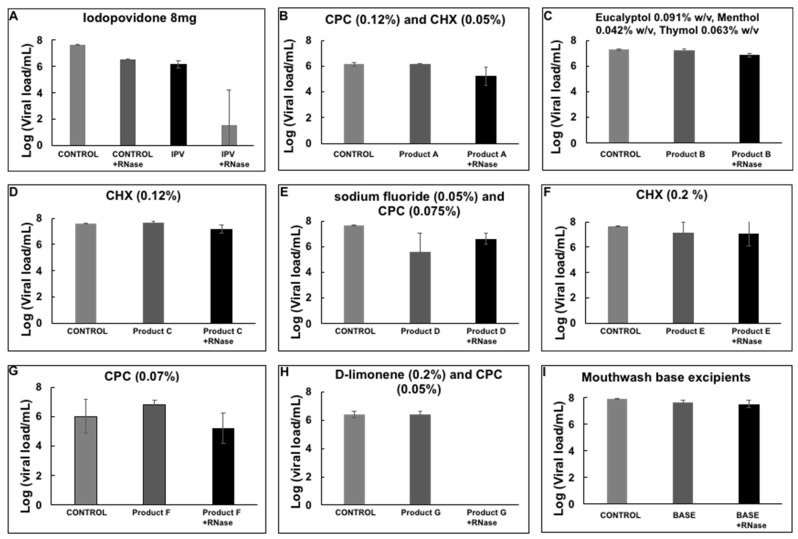
Viral load reduction of SARS-CoV-2 from nasopharyngeal samples by mouthwash products. (**A**) Iodopovidone *; (**B**) Product A **; (**C**) Product B ^††^; (**D**) Product C ^†††^; (**E**) Product D ^***^; (**F**) Product E ^‡‡‡^; (**G**) Product F ^‡‡^; (**H**) Product G ^§§^; (**I**) Mouthwash base excipients. Plots show the Mean and SD of 4–5 experiments.

There is a decrease in the amount of viral load in the RNase treated sample, presumably due to broken viral particles and naked RNA present in the swab sample that was degraded during the RNase treatment. However, most of the RNA was preserved and quantified. The assay was tested with an iodopovidone solution (IPV), previously reported to have strong virucidal activity against SARS-CoV-2 in in vitro experiments [29]. A 1-min incubation of IPV with the nasopharyngeal swab positive sample reduced the viral load completely, more than 6 logs reduction efficacy (Figure 3), suggesting that IPV degrades or compromises the integrity of the viral envelope, exposing the RNA to the RNase enzyme to degrade it, and presumably inactivating the virus. The virucidal activity of several commercial mouthwash products was subsequently tested by the RNA inactivating assay. We observed a reduction of about 6 logs with a D-limonene (0.2%) and CPC (0.05%) containing formula ^§§^.

However, none of the other solutions showed a reduction in viral load with the assay presented here (Figure 3).

**Figure 4:**
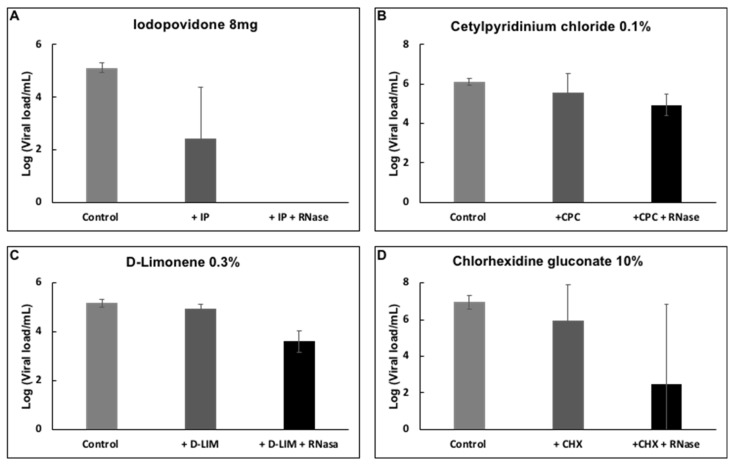
Effect of (**A**) IPV * 4%, (**B**) CPC ^†^ 0.1%, (**C**) D-limonene ^‡^ 10%, and (**D**) CHX ^§^ 10% on the detection of SARS-CoV-2 RNA by RT-PCR. Plots show the Mean and SD of 4–5 experiments.

Viral samples were incubated with D-limonene, CPC, or CHX, common compounds found in mouthwash solutions, to determine whether they had any impact on the reduction of viral load. After incubation with the different compounds already mentioned, D-limonene and CHX resulted in a reduction of viral load of about 1.5 logs (~95% reduction) (Figure 4). However, RNA was still present and resisted degradation through the RNase treatment.

## 4. Discussion

One of the main current concerns in dentistry is the risk of infection with SARS- CoV-2 while working with patients. A high risk of infection by SARS-CoV-2 while sharing settings that are enclosed, confined, or crowded, has also been described [30]. This has been increased due to new variants, as the Delta variant infections are on average ~1000 times greater compared to A/B lineage infections during the initial epidemic wave in China in early 2020, implying that Delta could replicate more quickly and be more contagious during the early stages of infection [31].

New alternatives of reducing the risk of infection by decreasing the viral loads of contagious individuals are being evaluated in different centers worldwide. Different protocols and recommendations have been published addressing key points to reduce this risk.

For dentists, using mouthwash before a dental appointment is one of the most promising ways to lower the risk of SARS-CoV-2 infection. Considering the way SARS-CoV-2 is transmitted, reducing its risk of infection has become a safety priority for dentists, as well as for the general population.

In May 2020, the Cochrane Library published a review with recommendations for the re-opening of dental services [32], reporting 82% of sources recommended the use of pre-operative mouthwashes at the dental office. This recommendation was issued without robust evidence, but other published articles do suggest that it is a recommendation that should be followed [33].

Mouthwashes directly inactivating SARS-CoV-2 would be crucial to creating a protocol to reduce the spread of COVID-19 [34].

Few articles have examined the inactivating effect of chlorhexidine gluconate (CHX) on SARS-CoV-2 [35,36], and some have suggested the effects of cetylpyridinium chloride (CPC) on other viruses [37], but there are currently no data on direct inactivation effects of CPC on SARS- CoV-2 [26]. On the other hand, previous studies on virucidal activities have been tested against some coronaviruses [38,39]. Benzalkonium chloride and hydrogen peroxide have also been tested against coronaviruses [37]. The relevance of using CDCM on day 1 (4 h after the initial dose) to reduce the SARS-CoV-2 viral load in saliva was supported by a multicenter, randomized, double-blind controlled trial of an antiviral mouthwash as a barrier measure in the SARS-CoV-2 transmission in people with asymptomatic to moderate COVID-19 symptoms; the mouthwash was commercially available with cyclodextrin and citrox (bioflavonoids) (CDCM) [40]. A randomized clinical trial found that mouthwash containing CPC + Zinc and CHX resulted in substantial reduction of the SARS-CoV-2 viral load in saliva for up to 60 min after rinsing, whereas mouthwash with hydrogen peroxide (HP) resulted in a significant reduction for up to 30 min [41]. According to a study using an anionic phthalocyanine derivative (APD) in a mouthwash protocol, the mechanical action of the mouthwash containing a chemical with antiviral activity against SARS-CoV-2 may minimize patient symptoms and virus spread [42].

Our study shows an important reduction of about 6 logs (or more than 99.99%) in the virucidal activity via a solution containing D-limonene and CPC. This finding is a strong advantage when compared to other mouthwashes included in the study.

This study showed the antiviral activity against SARS-CoV-2 of the following oral care products: iodopovidone (8 mg); * D-limonene, a terpene extracted from citrus peels (0.3%); ^†^ cetylpyridinium chloride (0.1%) (CPC); ^‡^ chlorhexidine gluconate (10%) (CHX); ^§^ a CPC (0.12%) and CHX (0.05%) containing formula; ** a formula containing essential oils; ^††^ a CPC containing formula (0.07%); ^‡‡^ a D-limonene (0.2%) and CPC (0.05%) containing formula; ^§§^ a solution containing sodium fluoride (0.05%) and CPC (0.075%); *** a solution containing CHX (0.12%); ^†††^ a CHX (0.2%) containing formula ^‡‡‡^.

However, only iodopovidone and a D-limonene (0.2%) and CPC (0.05%) containing formula have shown a significant reduction of SARS-CoV-2 viral load using the biochemical assay.

## 5. Conclusions

The data in this report were collected in vitro, and a further evaluation of the anti-SARS-CoV-2 activity of the products is required in vivo, as for every other mouthwash.

However, within the limitations of this study, we report that D-limonene and CPC in mouthwash preparation reduced the virucidal activity of SARS-CoV-2 by about 6 logs. This reduction in virucidal activity has not been demonstrated yet by any other commercially available mouthwash.

Hence, establishing a clinical protocol for dentists is suggested, where all patients to be treated rinse pre-operatively with a mouthwash containing both D-limonene and CPC to reduce the likelihood of infection with SARS-CoV-2 for dentists.

Reducing the risk of infection with SARS-CoV-2 has become a safety priority for the general population at large, and a similar protocol might prove an inexpensive way to help reduce the spread of SARS-CoV-2.

## Figures and Tables

**Table 1:** Solutions used in the study.

	Product	Name (Laboratory)	Active Ingredient
*	IPV	Isodine^®^ (Bussie)	Iodopovidone (8 mg)
†	D-limonene	D-Limonene	D-limonene (0.3%)
**‡**	CPC	Cetylpyridinium chloride	Cetylpyridinium chloride (0.1%)
**§**	CHX	Chlorhexidine gluconate	Chlorhexidine gluconate 10%
**	Product A	PERIO-AID^®^ (DENTAID)	CPC (0.12%) and CHX (0.05%)
**††**	Product B	Listerine^®^ Zero Alcohol (Johnson & Johnson)	Eucalyptol 0.091% *w*/*v*, Menthol 0.042% *w*/*v*, Thymol 0.063% *w*/*v*
**†††**	Product C	Periogard^®^ (Colgate)	CHX (0.12%)
***	Product D	Plax^®^ (Colgate)	Sodium fluoride (0.05%) and CPC (0.075%)
**‡‡‡**	Product E	Clorhexol^®^ (Farpag)	CHX (0.2%)
**‡‡**	Product F	Scope^®^ (P&G)	CPC (0.07%)
**§§**	Product G	Xyntrus^®^ (Brix Medical Science)	D-limonene (0.2%) and CPC (0.05%)
**§§§**	Mouthwash base excipients	Control reaction	Water, glycerin, citric acid, colorant, sodium citrate

## Abbreviations

*	Isodine^®^ Laboratorio Bussie.
†	Noncommercial mouthwash donated by Laboratorio Brix Medical Science.
‡	Noncommercial mouthwash donated by Laboratorio Brix Medical Science.
§	Noncommercial mouthwash donated by Laboratorio Brix Medical Science.
**	Perio-Aid^®^ Laboratorio Dentaid.
††	Listerine^®^ Zero Alcohol. Johnson & Johnson.
‡‡	Scope^®^ Procter & Gamble.
§§	Xyntrus^®^ Laboratorio Brix Medical Science.
***	Plax^®^ Colgate.
†††	Periogard^®^ Colgate.
‡‡‡	Clorhexol^®^ Laboratorios Farpag.

## References

[1] World Health Organization WHO Coronavirus Disease (COVID-19) Dashboard. https://covid19.who.int/.

[2] Zhu N., Zhang D., Wang W., Li X., Yang B., Song J., Tan W. (2020). A Novel Coronavirus from Patients with Pneumonia in China, 2019. N. Engl. J. Med..

[3] Zeidler A., Karpinski T.M. (2020). SARS-CoV, MERS-CoV, SARS-CoV-2 comparison of three emerging Coronaviruses. Jundishapur J. Microbiol..

[4] Liu J., Liao X., Qian S., Yuan J., Wang F., Liu Y., Wang Z., Wang F.S., Liu L., Zhang Z. (2020). Community Transmission of Severe Acute Respiratory Syndrome Coronavirus 2, Shenzhen, China, 2020. Emerg. Infect. Dis..

[5] Morawska L., Cao J. (2020). Airborne transmission of SARS-CoV-2: The world should face the reality. Environ. Int..

[6] van Doremalen N., Bushmaker T., Morris D.H., Holbrook M.G., Gamble A., Williamson B.N., Tamin A., Harcourt J.L., Thornburg N.J., Gerber S.I. (2020). Aerosol and Surface Stability of SARS-CoV-2 as Compared with SARS-CoV-1. N. Engl. J. Med..

[7] Wang W., Xu Y., Gao R., Lu R., Han K., Wu G., Tan W. (2020). Detection of SARS-CoV-2 in Different Types of Clinical Specimens. JAMA.

[8] Chang L., Zhao L., Gong H., Wang L., Wang L. (2020). Severe Acute Respiratory Syndrome Coronavirus 2 RNA Detected in Blood Donations. Emerg. Infect. Dis..

[9] (2020). Breastfeeding and COVID-19. World Health Organization: Geneva, Switzerland. https://www.who.int/news-room/commentaries/detail/breastfeeding-and-covid-19.

[10] Karia R., Gupta I., Khandait H., Yadav A., Yadav A. (2020). COVID-19 and its Modes of Transmission. SN Compr. Clin. Med..

[11] Andersen K.G., Rambaut A., Lipkin W.I., Holmes E.C., Garry R.F. (2020). The proximal origin of SARS-CoV-2. Nat. Med..

[12] Zhou P., Yang X.L., Wang X.G., Hu B., Zhang L., Zhang W., Si H.R., Zhu Y., Li B., Huang C.L. (2020). A pneumonia outbreak associated with a new coronavirus of probable bat origin. Nature.

[13] WHO (2020). Transmission of SARS-CoV-2: Implication for Infection Prevention Precautions. World Health Organization. https://www.who.int/news-room/commentaries/detail/transmission-of-sars-cov-2-implications-for-infection-prevention-precautions.

[14] Chia P.Y., Coleman K.K., Tan Y.K., Ong S.W.X., Gum M., Lau S.K., Marimuthu K. (2020). Detection of air and surface contamination by SARS-CoV-2 in hospital rooms of infected patients. Nat. Commun..

[15] Jiang Y., Wang H., Hao S., Chen Y., He J., Liu Y., Chen L., Yu Y., Hua S. (2020). Digital PCR is a sensitive new technique for SARS-CoV-2 detection in clinical applications. Clin. Chim. Acta.

[16] Liu Y., Ning Z., Chen Y., Guo M., Liu Y., Gali N.K., Sun L., Duan Y., Cai J., Westerdahl D. (2020). Aerodynamic analysis of SARS-CoV-2 in two Wuhan hospitals. Nature.

[17] Santarpia J.L., Rivera D.N., Herrera V.L., Morwitzer M.J., Creager H.M., Santarpia G.W., Crown K.K., Brett-Major D.M., Schnaubelt E.R., Broadhurst M.J. (2020). Aerosol and surface contamination of SARS-CoV-2 observed in quarantine and isolation care. Sci. Rep..

[18] Xu H., Zhong L., Deng J., Peng J., Dan H., Zeng X., Chen Q. (2020). High expression of ACE2 receptor of 2019-nCoV on the epithelial cells of oral mucosa. Int. J. Oral Sci..

[19] Ge Z.Y., Yang L.M., Xia J.J., Fu X.H., Zhang Y.Z. (2020). Possible aerosol transmission of COVID-19 and special precautions in dentistry. J. Zhejiang Univ. Sci. B.

[20] Meethil A.P., Saraswat S., Chaudhary P.P., Dabdoub S.M., Kumar P.S. (2021). Sources of SARS-CoV-2 and Other Microorganisms in Dental Aerosols. J. Dent. Res..

[21] Liu L., Wei Q., Alvarez X., Wang H., Du Y., Zhu H., Chen Z. (2011). Epithelial cells lining salivary gland ducts are early target cells of severe acute respiratory syndrome coronavirus infection in the upper respiratory tracts of rhesus macaques. J. Virol..

[22] To K.K., Tsang O.T., Yip C.C., Chan K.H., Wu T.C., Chan J.M., Leung W.S., Chik T.S., Choi C.Y., Kandamby D.H. (2020). Consistent detection of 2019 Novel Coronavirus in Saliva. Clin. Infect. Dis..

[23] Xu J., Li Y., Gan F., Du Y., Yao Y. (2020). Salivary glands: Potential reservoirs for COVID-19 asymptomatic infection. J. Dent. Res..

[24] Herrera D., Serrano J., Roldán S., Sanz M. (2020). Is the oral cavity relevant in SARS-CoV-2 pandemic?. Clin. Oral Investig..

[25] O’Donnell V.B., Thomas D., Stanton R., Maillard J.Y., Murphy R.C., Jones S.A., Humphreys I., Wakelam M.J.O., Fegan C., Wise M.P. (2020). Potential role of oral rinses targeting the viral lipid envelope in SARS-CoV-2 infection. Function.

[26] Seneviratne C.J., Balan P., Ko K., Udawatte N.S., Lai D., Ng D., Venkatachalam I., Lim K.S., Ling M.L., Oon L. (2021). Efficacy of commercial mouth-rinses on SARS-CoV-2 viral load in saliva: Randomized control trial in Singapore. Infection.

[27] Mateos Moreno M.V., Obrador A.M., Márquez V.A., Ferrer García M.D. (2021). Oral antiseptics against Coronavirus: In vitro and clinical evidence. J. Hosp. Infect..

[28] Corman V.M., Haage V.C., Bleicker T., Schmidt M.L., Mühlemann B., Zuchowski M., Jo W.K., Tscheak P., Möncke-Buchner E., Müller M.A. (2021). Comparison of seven commercial SARS-CoV-2 rapid point-of-care antigen tests: A single-centre laboratory evaluation study. Lancet Microbe.

[29] Anderson D.E., Sivalingam V., Kang A.E.Z., Ananthanarayanan A., Arumugam H., Jenkins T.M., Hadjiat Y., Eggers M. (2020). Povidone-iodine demonstrates rapid in vitro virucidal activity against SARS-CoV-2, the virus causing COVID-19 disease. Infect. Dis. Ther..

[30] (2020). Transmission of SARS-CoV-2: Implications for Infection Prevention Precautions. Scientific Brief. https://www.who.int/publications/i/item/modes-of-transmission-of-virus-causing-covid-19-implications-for-ipc-precaution-recommendations.

[31] Li B., Deng A., Li K., Hu Y., Li Z., Xiong Q., Liu Z., Guo Q., Zou L., Zhang H. (2021). Viral infection and transmission in a large, well-traced outbreak caused by the SARS-CoV-2 Delta variant. Medrxiv.

[32] Jan C., Craig R., Magaly A.-M., Miriam B., Thibault C., Manas D., Anne-Marie G., Beatriz G., Thomas L., Derek R. 16 May 2020 Recommendations for the Re-Opening of Dental Services: A Rapid Review of International Sources (Substantial Update 16 May 2020) Cochrane Database of Systematic Reviews. https://oralhealth.cochrane.org/news/recommendations-re-opening-dental-services-rapid-review-international-sources.

[33] Vergara-Buenaventura A., Castro-Ruiz C. (2020). Use of mouthwashes against COVID-19 in dentistry. Br. J. Oral Maxillofac. Surg..

[34] Muñoz-Basagoiti J., Perez-Zsolt D., León R., Blanc V., Raïch-Regué D., Cano-Sarabia M., Izquierdo-Useros N. (2021). Mouthwashes with CPC reduce the infectivity of SARS-CoV-2 variants in vitro. J. Dent. Res..

[35] Komine A., Yamaguchi E., Okamoto N., Yamamoto K. (2021). Virucidal activity of oral care products against SARS-CoV-2 in vitro. J. Oral Maxillofac. Surg. Med. Pathol..

[36] Meister T.L., Brüggemann Y., Todt D., Conzelmann C., Müller J.A., Groß R., Steinmann E. (2020). Virucidal efficacy of different oral rinses against severe acute respiratory syndrome Coronavirus 2. J. Infect. Dis..

[37] Baker N., Williams A.J., Tropsha A., Ekins S. (2020). Repurposing quaternary ammonium compounds as potential treatments for COVID-19. Pharm. Res..

[38] Kampf G., Todt D., Pfaender S., Steinmann E. (2020). Persistence of coronaviruses on inanimate surfaces and their inactivation with biocidal agents. J. Hosp. Infect..

[39] Khokhar M., Roy D., Purohit P., Goyal M., Setia P. (2020). Viricidal treatments for prevention of coronavirus infection. Pathog. Glob. Health.

[40] Carrouel F., Valette M., Gadea E., Esparcieux A., Illes G., Langlois M.E., Perrier H., Dussart C., Tramini P., Ribaud M. (2021). Use of an antiviral mouthwash as a barrier measure in the SARS-CoV-2 transmission in adults with asymptomatic to mild COVID-19: A multicentre, randomized, double-blind controlled trial. Clin. Microbiol. Infect..

[41] Eduardo F.P., Corrêa L., Heller D., Daep C.A., Benitez C., Malheiros Z., Stewart B., Ryan M., Machado C.M., Hamerschlak N. (2021). Salivary SARS-CoV-2 load reduction with mouthwash use: A randomized pilot clinical trial. Heliyon.

[42] da Silva Santos P.S., da Fonseca Orcina B., Machado R., Vilhena F.V., da Costa Alves L.M., Zangrando M., de Oliveira R.C., Soares M., Simão A., Pietro E. (2021). Beneficial effects of a mouthwash containing an antiviral phthalocyanine derivative on the length of hospital stay for COVID-19: Randomised trial. Sci. Rep..

